# The effect of avocado seed powder (*Persea americana* Mill.) on the liver and kidney functions and meat quality of culled female quail (*Coturnix coturnix japonica*)

**DOI:** 10.14202/vetworld.2019.1608-1615

**Published:** 2019-10-24

**Authors:** Elly Tugiyanti, Ning Iriyanti, Yosua Sujud Apriyanto

**Affiliations:** 1 Department of Animal Production, Faculty of Animal Science, University of Jenderal Soedirman, Purwokerto, Indonesia; 2 Department of Animal Nutrition, Faculty of Animal Science, University of Jenderal Soedirman, Purwokerto, Indonesia

**Keywords:** cooking loss, flavonoid, natural antioxidant, poultry

## Abstract

**Background and Aim:**

High temperatures have a detrimental effect on quail performance, even disrupting the immune system and function of the internal organs. This research aimed to investigate the effectiveness of avocado seed powder supplements on meat quality and the liver and kidney functions of culled female quails.

**Materials and Methods:**

A total of 100 six-month-old culled female quail were allotted to four dietary treatments, i.e., R0: Basal feed without avocado seed powder supplement and R1, R2, and R3 with basal feed + 3%, 6%, and 9% avocado seed powder supplement, respectively. The observed variables included meat quality (protein, fat, cholesterol and meat collagen, water holding capacity, and tenderness), liver function (liver weight, serum glutamic oxaloacetic transaminase [SGOT], and serum glutamic pyruvate transaminase [SGPT]), and kidney function (urea level, creatinine, uric acid, albumin, and glucose).

**Results:**

Analysis of variance showed that avocado seed powder supplements significantly affected the level of SGOT, urea, creatinine, protein, fat, cholesterol, meat tenderness, and cooking loss. A non-significant effect was found on liver weight, SGPT, uric acid, albumin and glucose blood level, collagen, or water holding capacity level.

**Conclusion:**

Avocado seed powder supplements improved meat quality as well as the liver and kidney functions of the culled female quail.

## Introduction

Indonesian people rely on livestock products such as poultry to meet protein demand [1]. Quail is popular food-producing livestock as it starts laying eggs at 42-45 days of age. Female quails are bred for their eggs until they stop lying and culled for the meat [2]. Quail belongs to the homeothermic group of animals commonly prone to stress which decreases performance [3]. Meanwhile, the average temperature in Indonesia is relatively high, at 23-36°C [4]. Heat stress potentially increases blood pressure leading to a disruptive blood function in transporting oxygen, nutrition and metabolism residue, and impaired immune system, and organ functions [5,6]. This condition triggers damage in liver and kidney which maintain detoxification and secretion during heatstroke [7,8]. The liver also secretes enzymes including serum glutamic oxaloacetic transaminase (SGOT) and serum glutamic pyruvate transaminase (SGPT) into the blood, reflecting a normal function. Kidney damage can be identified by examining the creatinine and blood glucose levels the indicators of kidney performance [9].

Plant-based antioxidants are gaining popularity [10]. Phytochemicals from summer shield, herbs, and flax and fenugreek seeds can reduce plasma concentrations of triglyceride (TG), total cholesterol, high-density lipoprotein (HDL)-cholesterol, low-density lipoprotein (LDL)-cholesterol, glutamic oxaloacetic transaminase (GOT), glutamic pyruvic transaminase (GPT), glucose, and glutathione peroxidase; however, TGs, plasma HDL, luteinizing hormone secretion, follicle-stimulating hormone, and estradiol-17β may increase [11-13]. Avocado (*Persea americana* Mill.) grows well in tropical areas such as Indonesia. In 2017, there were 2,312,145 avocado trees in the country, producing 363,157 tons of fruit, 106,426 kg of which were for export [14]. A 0.3-0.5 kg fuerte avocado consists of 73.16±0.28% flesh, 15.02±0.08% seed, and 11.82±0.32% skin [15]. Only the flesh part is used and waste the seed which is a renowned source of carotenoids, minerals, phenolics, vitamins, fatty acids, saponin, tannin, flavonoid, glycoside, cyanogenic, alkaloid, phenol, and steroids [16,17]. The flavonoid in an avocado seed is a water-soluble phenolic compound with antioxidant properties [18]. The avocado seed has been reported to be rich in polyphenols, with antimicrobial and antioxidant activities [18]. It contains 150.6-265.75 mg ascorbic acid equivalents (AAE)/100 g antioxidant, 1.92 mg quercetin 100 g flavonoid [19], and >70% phenolic [17]. The phenolic compound in an avocado seed is 44.89 mg/kg, higher than that in the leaves or flesh [15,17,18]. Herbal flavonoids activate appetite mechanisms in poultry, contribute to balancing the gastrointestinal microflora system, and exhibit anti-inflammatory and antioxidant effects. Phenol, flavonoids, and the scavenging ability of *P. americana* are positively correlated because flavonoids and phenol are antioxidants that inhibit lipid peroxidation, and reduce oxidative stress [19].

The natural antioxidants effects in avocado seed powder on quail have not been investigated elsewhere when this paper is being written. In general, research into the effect of natural antioxidants focus on increasing production, performance and immune levels, but the negative influence on the liver and kidney functions of quails has never been examined. This research aimed to investigate the effectiveness of avocado seed powder supplements on the liver and kidney functions and meat quality of culled female quail.

## Materials and Methods

### Ethical approval

The current study was carried out as a part of the Institute Research Project, and has been approved by the Research Committee.

### Animals and feed

A total of 100 6-month-old culled female quail were raised at the research farm in Sokaraja Kulon Village, Banyumas District. Sokaraja Kulon is situated on the latitude 7° 19’ 30” S and longitude 108° 39’ 20” E with hot and humid weather around 25.6-34.2°C. The culled female quails were randomly allocated into 20 cages with five quail per cage. Feed was given *ad libitum* twice a day at 7 am and 3 pm, and water was freely accessible. Quails’ health status was controlled daily, and weight was measured on a weekly basis.

The feed for 10-week experiment was made of avocado seed powder, corn, rice bran, fishmeal, soy waste, palm oil, Topmix, salt, lysine, and methionine. The feed composition and formulation are shown in Table-1.

**Table 1:** Treatment feed formulation.

Feed components	Avocado seed powder supplement (%)			

	0	3	6	9
Corn	45	45	45	45
Rice bran	27.50	27.50	27.50	27.50
Soy waste	15	15	15	15
Fishmeal	8	8	8	8
Avocado seed powder	0	3	6	9
Oil palm	1.20	1.20	1.20	1.20
Calcium carbonate (CaCO_3_)	2.50	2.50	2.50	2.50
Premix	0.20	0.20	0.20	0.20
Lysine	0.20	0.20	0.20	0.20
Methionine	0.40	0.40	0.40	0.40
Total	100	103	106	109
Nutrient content				
CP (%)	18.28	18.59	18.90	19.21
ME (kcal/kg)	2862.52	2969.63	3076.72	3183.85
CFat (%)	5.81	5.98	6.16	6.33
CF (%)	4.48	4.66	4.84	5.03
Ca (%)	2.76	2.78	2.80	2.82
P (%)	1.25	1.25	1.26	1.27
Lysine (%)	1.12	1.12	1.12	1.12
Methionine (%)	0.71	0.71	0.71	0.71

The result of proximate analysis in Laboratory of Livestock Nutrition, Animal Science Faculty, Jenderal Soedirman University and LPPT UGM (2019)

The culled quail was let in the 1-week preliminary before treatment. Avocado seed powder supplement was offered gradually across 4 days. Day 1 was 25% treatment feed +75% commercial feed, Day 2 was 50% treatment feed +50% commercial feed, Day 3 was 75% treatment feed +25% commercial feed, and Day 4 onward 100% treatment feed. The avocado seed powder was mixed with basal feed according to the dietary treatment level; i.e., 0, 3, 6, and 9% for 3 months, from November 2018 to January 2019.

The quails were weighed (163-168 g) before blood collection through venipuncture, the quails were slaughtered.

### Experimental design

The research was conducted by a completely randomized design [20]. The four dietary treatments consisted of R0: Basal feed without avocado seed powder supplement, R1: Basal feed +3% avocado seed powder supplement; R2: Basal feed +6% avocado seed powder supplement; and R3: Basal feed +9% avocado seed powder. Each treatment was repeated 5 times to achieve 20 treatment units, each with five quail. The observed variables included meat quality (protein, fat, cholesterol, and meat collagen, water holding capacity, and tenderness), liver function (SGOT and SGPT), and kidney function (urea level, creatinine, and uric acid).

### Avocado seed powder production

The avocado seed powder was made by soaking the avocado seed in water for 12 h. It was then boiled, skinned, and thinly sliced for a quick sun-drying; subsequently, the dried seed was pulverized using a grinder. Proximate analysis showed that the avocado seed powder contained 11.83% water and 88.17% dry matter which consisted 5.77% crude protein, 7.33% crude fat, 3.14% fiber, 2.71% ash, 81.04% nitrogen-free extract, and 3881.13 kcal/kg metabolic energy. The phytochemical compounds in the avocado seed powder were 5.54% tannins, 7.59% saponins, and 5.41% flavonoids.

### Analysis of liver and kidney functions

The blood samples were taken to observe the liver and kidney functions 1 day before the quails­ were slaughtered. The observed parameters included SGOT and SGPT enzymes, urea, creatinine, and uric acid, using an autoanalyzer (Reflotron [R] plus) [21]. One drop of blood (30 μl) was smeared on a slide. Each parameter used different slides inserted into the autoanalyzer. The results were shown after a few minutes.

### Analysis of meat cholesterol

Analysis of meat quality included meat cholesterol, protein, fat, collagen, tenderness, cooking loss, and water holding capacity. Cholesterol level was analyzed using the Liebermann–Burchard method [17]. Chloroform extract that contained cholesterol would react with acetic anhydride acid and thick sulfate acid and form a colored reaction.

### Analysis of meat quality

The physical properties of the meat (tenderness, cooking loss, and water holding capacity), collagen, fat, water content, and protein level were analyzed using Foodscan™ with Near Infra-Red performance [22]. The samples were pulverized using a meat grinder, then weighed ±100 g and placed in a sample cup (15 cm diameter) to the brim. At this stage, the computer was turned on and connected to the food scan. The food scan icon was pressed to activate the food scan program. The sample cup was inserted to the compartment and the “tool analysis” icon was turned on (to detect the average water content, protein, fat, and collagen in percentage unit/%). Each sample was coded, and the results were printed out.

### Statistical analysis

The obtained data were subjected to analysis of variance, and any significant effects would be employed to an Honestly Significant Difference (HSD) test.

## Results

### Liver function

Liver weight and the SGPT and SGOT levels of the culled female quail given feed supplements with avocado seed powder were within the normal range (Table-2), similar to those in a report by Ardiani [23]. Therefore avocado seed powder supplement of up to 9% did not harm the liver function of the culled female quail.

**Table 2:** The average level of SGPT and SGOT of culled female quail.

Avocado seed powder (%)	SGPT (mg/dL)	SGOT (mg/dL)	Liver weight (g)
0	31.78±4.74	81.56±12.74^a^	4.04±0.73
3	24.98±7.35	55.91±13.04^ab^	3.79±0.37
6	25.49±8.41	40.75±16.66^b^	3.75±0.26
9	29.31±10.85	48.93±18.48^b^	3.99±0.63
SEM	1.41	4.45	0.11
p	0.517	0.004**	0.779

a,b Means bearing different superscripts in a column differ significant (p<0.05). SEM: Standard error of mean. SGPT=Serum glutamic pyruvate transaminase, SGOT=Serum glutamic oxaloacetic transaminase

Table-2 shows that supplementing avocado seed powder to culled female quails did not significantly affect liver weight or SGPT, but it highly significantly affected (p<0.01) the SGOT level. About 6% supplement resulted in the lowest liver weight and SGPT level, but 3% supplement displayed similar SGOT and lower SGPT to the control group. Therefore, supplementing feed with 6% avocado seed powder improved the liver function of the quail.

### Kidney function

Quails fed with avocado seed powder supplement demonstrated normal kidney function with 5.45-10.27 mg/dL urea, 1.49-2.66 mg/dL creatinine, 3.60-4.32 mg/dL uric acid, 2.83-3.62 mg/dL albumin, and 89.80-1104.20 mg/dL glucose (Table-3). It was similar Ebile *et al*. [24] and Agina *et al*. [25]; i.e. 6.98-9.32 mg/dL, 0.35-0.44 mg/dL, and 1.08-5.47 mg/dL for normal urea, creatinine, and albumin, respectively. However, the uric acid was lower than those obtained by Agina *et al*. [25] at 16.02 mg/dL and glucose levels were lower than obtained by Scholtz *et al*. [26] at 14.4±0.6 mmol/L.

**Table 3:** The average level of urea, creatinine, and uric acid in culled female quail.

Avocado seed powder (%)	Ureum (mg/dL)	Creatinine (mg/dL)	Uric acid (mg/dL)	Albumin (mg/dL)	Glucose (mg/dL)
0	10.27±2.01^a^	2.66±0.60^a^	3.60±0.89	2.83±0.75	93.40±15.07
3	6.24±1.19^b^	1.52±0.46^b^	4.32±1.12	2.90±1.12	99.80±10.18
6	6.19±1.43^b^	1.49±0.37^b^	4.24±1.21	3.21±1.55	89.8±13.14
9	5.45±2.34^b^	1.73±0.42^b^	4.28±1.14	3.62±1.78	104.20±15.54
SEM	0.21	0.14	1.42	0.29	3.08
p	0.003**	0.004**	0.697	0.788	0.373

a,b Means bearing different superscripts in a column differ significant (p<0.05). SEM: Standard error of mean

Analysis of variance showed that the avocado seed powder supplement significantly affected (p<0.01) urea and creatinine levels, but not uric acid, albumin, and glucose levels. The HSD test indicated that the control group had different results from the 3%, 6%, and 9% supplement groups, as the flavonoids in avocado seed powder improved the health status of laboratory animals and prolong the shelf life of poultry products [27].

### Meat quality of the culled quail

The protein level of the quail that consumed feed supplemented with avocado seed powder was 21.38-23.56% (Table-4). It was higher than 17% [28] but relatively similar to that of broiler quail, i.e., 19.91-22.72% [29] and 21.65% [30]. Meat quality is assessed from its chemical and physical properties. Quails, chicken and beef share similar protein content (21%), fat and cholesterol levels. However, tenderness is the ultimate quality of quail. Animal meat would get tougher and contained higher collagen as it gets older [31]. Analysis of variance showed that supplementing feed with avocado seed powder significantly (p<0.01) increased the meat protein of the quails compared to those fed with basal feed.

**Table 4:** Nutrient content of culled quail meat.

Avocado seed powder (%)	Protein (%)	Fat (%)	Cholesterol (mg/100 g)	Collagen (%)
0	21.38±0.23^a^	5.17±0.19^a^	39.68±4.70^a^	1.89±0.06
3	21.8±0.87^a^	4.67±0.08^b^	31.15±1.38^b^	1.89±0.15
6	23.56±0.36^b^	5.29±0.40^a^	29.16±3.03^bc^	1.86±0.08
9	22.72±1.18^ab^	5.11±0.18^ab^	24.56±1.78^c^	1.91±0.09
SEM	0.25	0.07	1.40	0.02
p	0.002**	0.006**	0.000**	0.826

a,b,c Means bearing different superscripts in a column differ significant (p<0.05). SEM: Standard error of mean

The fat and cholesterol content of the quail fed on the avocado seed powder supplement was between 4.67-5.17% and 24.56-39.68 mg/100 g, respectively (Table-4). Meat fat in this study was higher than 3.598-4.584% of the previous study [32], but the cholesterol level was lower than 10.00 µg/g [33]. Analysis of variance showed that the avocado seed powder significantly (p<0.01) affected fat content and lowered cholesterol level (p<0.01) (Table-4). The meat fat of the culled female quail fed on control feed (0%) was not significantly different from that of the 6% and 9% supplementation. However the lowest, meat fat was observed in the 3% supplement group.

The collagen level of the quail after avocado seed powder treatment was 1.86-1.89% (Table-4), similar to 1.70-2.28% with Azolla microphylla supplement [32]. Analysis of variance showed that avocado seed powder supplementation did not significantly affect the collagen level of the quail. About 3% and 6% avocado seed powder could reduce the collagen level.

## Discussion

The liver is an organ that plays a crucial role in regulating most body metabolisms, such as carbohydrate, protein, and fat as well as synthesizing (excretion) of some protein parts, blood coagulation, urea, and other vital substances in the body. In addition, most cholesterol is produced in the liver [34]. It is where bile acid is formed and transported, and where steroid hormones (estrogen) are degraded. The essential function of the liver is to filter and discharge toxins in the body [35].

GOT is an enzyme in the cells of liver and other organs that will be discharged into the blood when the liver is damaged. GPT serum is the dominant enzyme in the liver. In SGOT and SGPT tests, the liver is considered damaged when the amount of enzyme in the plasma surpasses the standard. SGPT is a more sensitive indicator than SGOT because it is only present in hepatocyte and in low concentrations in other tissues.

Avocado seeds display antioxidant activity because they contain carotenoids, minerals, phenolics, vitamins, fatty acids, saponins, tannins, flavonoids, glycoside, alkaloids, phenols, and steroids [16,17]. These active compounds tend to reduce SGOT and SGPT, maintaining the liver weight. Avocado seeds are rich in Vitamin C and antioxidant by converting α-ocopheroxyl radicals to α-tocopherol. Flavonoids and phenols are reported to prevent liver damage [19].

Avocado seed powder contains active substances such as flavonoids, quercetin, vitamins, and minerals. Tanin inhibits fat absorption in the intestine, quercetin reduces accumulated fat molecules, and flavonoid and polyphenol increase lipase lipoprotein activity to prevent hyperlipidemia which triggers diseases such as liver damage. In other words, propolis can protect the internal organs, especially the liver [36]. Natural polyphenol is a plant-based secondary metabolite with a pharmacological effect on oxidative stress, lipid metabolism, insulin resistance, and inflammation, and encompasses the most crucial pathological process in the etiology of liver disease [35].

Polyphenol can suppress gene expression related to distressed cells and manage the cells involved in bile acid synthesis, elongated unsaturated fatty acid, and tetrahydrofolate synthesis [36]. Flavonoids comprise a diverse group of compounds found in various human foods. Flavonoid intake has been associated with several health benefits due to its potential antioxidant activity. Moreover, further research has revealed other activities such as anti-inflammatory and metabolic effects. Flavonoids display a multitude of activities; therefore, these compounds have been upgraded from antioxidant to bioactive compounds [37]. Some flavonoids, such as catechin, apigenin, and quercetin exhibit hepatoprotective activity and antihepatotoxic properties because the compounds inhibit lipid peroxide in the cell membrane. Quercetin exhibits antioxidant activity by inhibiting inflammation enzyme activities or by increasing glutathione synthesis [38]. Flavonoids are reported to stimulate the activity of RNA polymerase 1 DNA-dependent and biosynthesis of RNA and protein, resulting in DNA biosynthesis and cell proliferation, which lead to regeneration in the damaged part of the liver [23].

Urea and creatinine are chemical compounds to indicate normal kidney function, while creatinine is the result of endogen metabolism – the yield of phosphocreatine degradation to evaluate the glomerulus function. Urea is the final product of protein and amino acid catabolism in the liver distributed through intracellular and extracellular fluid into the blood and filtrated by the glomerulus. Uric acid is the product of purine nucleic acid catabolism, although the glomerulus filtrates the uric acid and secretes it by tubules distal, which is discharged in the feces [9]. Table-3 shows that the creatinine level fell from 2.66±0.60 to 1.49±0.37 mg/dL due to flavonoid compounds. In degrading calcium oxalate crystals, flavonoid bonds with calcium to form a Ca–flavonoid complex compound. Furthermore, flavonoid inhibits urolithiasis by disrupting the production of reactive oxygen species (ROS), thus preventing oxidative stress that leads to cell damage such as kidney obstruction [39].

In addition, the saponin, alkaloids, flavonoids, polyphenol, quercetin, and alcohol glucose of avocado seed powder contain amino acid (tryptophan and lysine), calcium, iron, tannin, sulfur, Vitamin A, Vitamin B, and Vitamin C, which can increase the glomerulus filtration rate, and inhibit the increase of urea and creatinine. Moreover, these compounds can inhibit urine crystallization and reduce lesion percentage in the kidney. Flavonoid lowers uric acid by inhibiting the action of oxidase xanthine enzyme in preventing or reducing the production of uric acid. Some flavonoids also serve as an antioxidant against superoxide radicals. Creatinine is the product of muscle metabolism; therefore, increasing creatinine levels signify kidney damage [40]. Furthermore, kidney failure is related to blood glucose level, as hyperglycemia plays a role in forming atherosclerosis, which narrows the lumen of the blood vessels and slows blood circulation rate down, leading to depletion of blood supply to the kidneys. It may cause a disturbance in the filtration process in the glomerulus and weaken kidney functions [39].

Albumin is a major protein in the blood that significantly determines the transport of physiological materials or body metabolites such as fatty acids, hormones, bilirubin, and external ligands, as well as the regulatory system of colloidal osmoses blood pressure. Avocados are loaded with antioxidants, phytochemicals, vitamins, minerals, fiber, and monounsaturated healthy fats [41]. Avocado seed flour did not affect blood albumin levels because the polyunsaturated fatty acids (PUFA) and monounsaturated fatty acid (MUFA) in avocado seed flour hydrolyzed by the enzyme lipase into free fatty acids (FFAs) in the blood could not quantitatively stimulate albumin synthesis in the liver. FFAs with the C4-C12 chain in blood plasma are always bound to albumin. It was in line with Murray *et al*. [42] that albumin is able to bind various types of ligand such as FFA.

In this study, blood glucose level was very low. As the quail gets mature, the erythrocytes count increases, but the blood glucose concentration decreases [43], presumably due to the unsaturated fatty acids, such as linoleic acid and oleic acid in the avocado seed flour. Linoleic acid includes unsaturated fatty acids with two double bonds PUFA, and oleic acid is an unsaturated fatty acid with one double/single bond MUFA. Diets high in unsaturated fatty acids in feed can increase the fluidity of cell membranes; therefore, the insulin receptors and glucose used by cells increase. The fatty acid composition of the lipid membrane affects the insulin action. Diets high in unsaturated fatty acids could increase membrane fluidity due to the increased insulin secretion and glucose used by cells [44]. Flavonoids increase glycolysis and glycogen pathways by suppressing the glycogenolysis and gluconeogenesis pathways, decrease glucose level [45].

The flavonoid in avocado contains high antioxidant properties [18], inhibits protein denaturation (the loss of structure and function of protein due to stress) and prevents the free radical formation in the body [46]. In addition, phytochemicals in avocado seed powder can increase acidic conditions in the pancreas, duodenum, and gallbladder, thereby increasing protease enzyme activity in breaking protein into amino acids [47]. Amino acids will be absorbed and converted into protein that consists of myofibrillar, sarcoplasmic, and connective tissue [48].

Avocado seed powder contains chemical compounds that are secondary metabolites, such as essential oil, alkaloids, flavonoids, saponins, and tannins, which can lower cholesterol levels. Flavonoid is an antioxidant that can lower fat and cholesterol in the blood, and saponin can lower cholesterol because it forms a complex bond that is insoluble in cholesterol. Several studies had shown that saponins from different sources exhibits lower serum cholesterol levels [1,49]. Besides acting as an antioxidant, polyphenol lowers cholesterol, LDL, and TG by improving the activity of lipase lipoprotein, resulting in a higher TG-enriched lipoprotein or a very-low-density lipoprotein (VLDL) and intermediate-density lipoprotein (IDL). The increasing HDL cholesterol level is partly due to the decreased VLDL level or the increased production of apolipoprotein (Apo) AI and Apo AII. A depleted LDL level may due to the increased VLDL and IDL clearance in the liver; therefore, LDL production declines [50]. Tannin could inhibit cholesterol by forming a reaction with mucosa protein and epithelial cells, which inhibits fat absorption [49].

Collagen is the most fibrous protein in animals, including quail; it contains about 33% of glycine and 21% of proline. Precursor collagen, trophagenagen, is a triple helix of three polypeptides chains, joined by hydrogen bonds. Avocado seed flour contains various active compounds, such as flavonoids and saponin, which display anti-inflammatory activity. Saponins inhibit the work of the cyclooxygenase enzyme by catalyzing the reaction of arachidonic acid to endoperoxidase. The inhibited cyclooxygenase and lipoxygenase enzymes shorten, the inflammatory reaction, and expedite the ability of transforming growth factor-B (TGF-B). TGF-B plays a role in stimulating fibroblast migration and proliferation. The fibroblast synthesizes collagen by forming new connective tissue, thus increasing the number of fibroblast cells and the collagen fibers subsequently [51].

Avocado flour supplementation did not affect collagen synthesis because the oxygen significantly affects the fiber preparation process, where the more oxygen presents, the more likely ROS is produced. ROS is crucial for physiology, but it grows in a pathological condition which could damage cells. In general, the mechanism of collagen reduction is correlated with the increased protease enzyme activity that reduces collagen production, increases collagen degradation, and accumulates the extracellular matrix of elastin [52].

Collagen fibers are the main fibers from connective tissue throughout the quail body. They are the main protein of the extracellular matrix, which contributes to the tenderness of meat. In line with Tugiyanti and Herijanto[32], the factors influencing meat tenderness are correlated with its composition, including fat cells between the meat fiber and collagen. The avocado seed has been reported to be rich in polyphenols, displaying antimicrobial, and antioxidant activities [18]. One seed contains 150.6-265.75 mg AAE/100 g antioxidant, and 1.92 mg quercetin/100 g flavonoid [19], and >70% phenolic [17]. The Vitamin C contained in avocado seed flour has a stimulating effect on increasing mRNA from collagen Types I and III. Vitamin C helps collagen synthesis and work as a cofactor in the hydroxylation process to activate prolyl hydroxylase to convert procollagen into collagen and lysis hydroxylase to crosslink to obtain a strong triple helix. In this study, avocado seed flour supplementation could influence collagen levels because the oxidation of Vitamin C with Fe^2+^ cofactors released many superoxide (O_2_-) oxygen anions/radicals. When O_2_- production is higher than the available oxygen, collagen synthesis increases. Avocado flour contains polyphenols and flavonoids exhibiting antioxidant activity, so O_2_- can be neutralized and collagen synthesis remains stable [53].

Compared to a previous study [54], this research reported similar water holding capacity (35.88-36.63% vs. 36.38-37.33%) but a lower cooking loss (22.79-25.21% vs. 31.99-40.21%). Analysis of variance showed that avocado seed powder supplementation significantly affected meat tenderness (p<0.01) and cooking loss (p<0.05), but not on water holding capacity (Table-5). Therefore, the meat fat was low and it slightly increased water holding capacity because of the intramuscular fat store [55]. The flavonoid effect of avocado seed powder could prevent meat fat oxidation due to oxidative stress [56-58]. Furthermore, the natural antioxidant actively promoted meat protein degradation, which made the meat more digestible, while maintaining physical meat properties as the indicators of meat quality by inhibiting the rate of oxidative damage.

**Table 5:** The quality physical properties of meat.

Avocado seed powder (%)	Water holding capacity	Cooking loss	Tenderness (kg/cm^2^)
0	36.21±0.94	24.21±0.57^a^	4.79±0.30^a^
3	36.63±0.70	23.03±0.66^ab^	4.04±0.10^b^
6	36.50±0.66	23.43±0.50^ab^	3.97±0.30^b^
9	35.88±1.28	22.79±1.13^b^	4.03±0.56^b^
SEM	0.20	0.20	0.10
p	0.601	0.045	0.006

a,b Means bearing different superscripts in a column differ significant (p<0.05). SEM: Standard error of mean

## Conclusion

Avocado seed powder supplement improves meat quality, and the liver and kidney functions of culled female quail.

## Authors’ Contributions

YSA performed the experiment under the supervision of ET and NI. NI designed the concept, and analyzed, and interpreted the data. ET did the proofreading, guided the preparation, and finalized the manuscript for publication. All the authors read and approved the final manuscript.
